# Effects of Dietary Energy Levels on Rumen Fermentation, Gastrointestinal Tract Histology, and Bacterial Community Diversity in Fattening Male Hu Lambs

**DOI:** 10.3389/fmicb.2021.695445

**Published:** 2021-09-10

**Authors:** Qiye Wang, Yutong Zeng, Xianglin Zeng, Xin Wang, Yancan Wang, Chunpeng Dai, Jianzhong Li, Pengfei Huang, Jing Huang, Tarique Hussain, Mingzhi Zhu, Huansheng Yang

**Affiliations:** ^1^Hunan Provincial Key Laboratory of Animal Intestinal Function and Regulation, Hunan International Joint Laboratory of Animal Intestinal Ecology and Health, Laboratory of Animal Nutrition and Human Health, College of Life Sciences, Hunan Normal University, Changsha, China; ^2^Hubei Zhiqinghe Agriculture and Animal Husbandry Co., Ltd., Yichang, China; ^3^Animal Sciences Division, Nuclear Institute for Agriculture and Biology, Faisalabad, Pakistan; ^4^National Research Center of Engineering Technology for Utilization of Functional Ingredients from Botanicals, Co-Innovation Center of Education Ministry for Utilization of Botanical Functional Ingredients, College of Horticulture, Hunan Agricultural University, Changsha, China; ^5^Hunan Provincial Key Laboratory of Animal Nutritional Physiology and Metabolic Process, Key Laboratory of Agro-Ecological Processes in Subtropical Region, Hunan Provincial Engineering Research Center of Healthy Livestock, Scientific Observing and Experimental Station of Animal Nutrition and Feed Science in South-Central, Ministry of Agriculture, Institute of Subtropical Agriculture, Chinese Academy of Sciences, Changsha, China

**Keywords:** rumen, bacteria, volatile fatty acid, high-throughput sequencing, Hu sheep

## Abstract

This study investigated rumen fermentation and histological and microbial diversity in male Hu lamb fed diets with different metabolizable energy (ME) levels (MEA, 9.17 MJ/kg, MEB, 10.00 MJ/kg, and MEC, 10.82 MJ/kg). Thirty-six male Hu lambs were randomly allotted to three treatments, and the feeding trial lasted for 67 days. Rumen fermentation results suggest that the iso-valerate had a significant effect on dietary energy level. The papillary height (PH) of rumen was the highest in the MEB group, the crypt depth (CD) was significantly increased in the duodenum and jejunum, and the villus height (VH)-to-CD ratio (VH/CD) was significantly decreased in the duodenum by increasing dietary energy levels; the VH, villus width (VW), and VH/CD also had significant differences in the ileum. 16S rRNA sequencing results showed that the operational taxonomic units (OTUs) number, the ACE, and Chao1 indices were linearly decreased by increasing dietary energy level; 24 phyla including 124 genera were identified, and the relative abundance of *Papillibacter* and *Quinella* linearly decreased by increasing the dietary energy level. Compared to MEA and MEB groups, the relative abundance of *unidentified_Veillonellaceae* and *Anaerovibrio* was significantly increased in the MEC group at the genus level. The relative abundance of the carbohydrate metabolism pathway predicted by Phylogenetic Investigation of Communities by Reconstruction of Unobserved States (PICRUSt) was linearly increased by increasing the dietary energy levels. Three metabolic pathways identified in Kyoto Encyclopedia of Genes and Genomes (KEGG) level 3 were significantly influenced as the dietary energy level increased. In summary, these results demonstrated that the dietary energy levels affected the rumen fermentation parameters, morphological structures of the gastrointestinal tract (GIT), and the composition and function of rumen microflora in male Hu sheep.

## Introduction

The Hu sheep is a unique breed native to China, and is one of the several white lamb-skin sheep breeds in the world (Wang et al., 2020a). Commonly, Hu sheep are identified as an ideal choice for factory production in our country, especially in southern agricultural areas (Wang et al., 2020b). With the deterioration of ecological environments and the increasing demand for consumption of lamb, ruminant production with traditional grazing patterns is dominated instead by large-scale farming with stall-feeding. As ruminant farming patterns have transformed, animal diets are witnessing a change. Furthermore, performance traits such as daily gain and rumen micro-ecosystem have changed accordingly (Morand-Fehr et al., 2007; Wang H. R. et al., 2017).

The rumen of ruminants is a dynamic and complex microecosystem that consists mostly of bacteria, protozoa, archaea, and fungi (Mackie et al., 2000; Knoell et al., 2016). Bacteria are the dominant microorganism group (Pitta et al., 2016), and some of these microorganisms that are attached to feed particles participate in the transformation of plant ingredients into animal products (Preston and Leng, 1987; Han et al., 2015). Rumen bacterial diversity is influenced by diet, breed, host age, season, and geographic region (Lee et al., 2012), and diet is a key factor in determining its composition and function (Wang et al., 2020b). The energy and protein of dietary nutrition are the two determinants that most often limit microbial activity (Clark, 1975; Clark and Davis, 1980). For instance, protein-degrading bacteria can degrade true proteins to volatile fatty acids (VFAs) and ammonia, and the major source of nitrogen for microbial growth and protein synthesis is ammonia (Wang et al., 2015; Owens and Basalan, 2016). Bach et al. (2005) found that a diet of high energy level can promote the synthesis of microbial proteins due to it supplying only enough available energy for microbial growth and metabolism (Owens and Basalan, 2016). Carbohydrates are a predominant energy source for ruminants, which generally make up more than 70% of the ruminant diet. The rumen is the main site for carbohydrate digestion as dietary carbohydrates are fermented by rumen microbes into VFAs (such as acetate, propionate, and butyrate), which can provide nearly two-thirds of the energy requirements for the host and further digested and absorbed by the gastrointestinal tract (GIT) (Cunha et al., 2011; McGovern et al., 2018). Although the concentration of dietary energy is important for rumen microbial growth and protein synthesis, excessive energy intake not only increases the cost of feed but may also lead to a decrease in meat quality (Broderick, 2003). Thus, it is necessary to explore appropriate dietary energy levels for ruminants.

Applying high energy (or high grain, such as corn) diet to promote animal performance was considered as a popular strategy in modern factory fattening systems of ruminants. Furthermore, to elucidate the GIT histology and rumen microbial community diversity and function, appropriate high-energy (or high-grain) diets are very urgent, especially for improving animal performance in large-scale healthy farming. Several recent studies have focused on rumen microbial diversity associated with dietary nutrition levels in goats and bulls (Zhang et al., 2017; McGovern et al., 2018) and high-grain diets in sheep (Seddik et al., 2019). However, so far, systematically conducted studies on the dynamic effects of dietary energy levels on rumen microbiota and GIT histology of sheep have not been reported. Here, we hypothesize that the rumen fermentation, GIT histology, and ruminal microbiota would dynamically alter by increasing the dietary energy levels in fattening male Hu lambs. Therefore, the present study was conducted to explore the dynamic variation in rumen fermentation, GIT histology, and rumen microbial community and function in Hu sheep with different dietary energy levels.

## Materials and Methods

### Animals, Diets, and Sampling Procedures

This experiment was performed in Hubei Zhiqinghe Agriculture and Animal Husbandry Co., Ltd., Yichang, Hubei, China. Thirty-six male Hu lambs (aged 4 months) with an average initial body weight (IBW) of 20.16 ± 0.38 kg were used in feeding trial. The lambs were allotted to one of three dietary treatments (groups MEA, MEB, and MEC), randomly based on their IBW. Each group included 12 lambs and fed in individual pens. All of the experimental diets met the nutritional requirements [National Research Council (NRC), 2007] for growing sheep with equivalent nutritional ingredients, except for energy. The dietary energy levels respectively were 9.17, 10.00, and 10.82 MJ/kg corresponding to 91, 99, and 107% of the energy levels that had been recommended by the Nutrient Requirements of Small Ruminants [National Research Council (NRC), 2007] for groups MEA to MEC, and the protein levels were all approximately 13% (Table 1). The experimental lambs feeding and management were referenced by Wang et al. (2020a); the trail lambs were fed their corresponding diets twice a day in the morning and afternoon and were subjected to free feeding and automatic drinking throughout the course of the experiment. At the end of the trial, five lambs close to the average weight from group MEA, group MEB, and group MEC, respectively, were selected for slaughter after they were not fed for 12 h, with a total 15 lambs being exsanguinated according to veterinary police rules. The rumen was removed from each of the sheep, and 100 ml of rumen fluid was homogenized and then divided into two 50-ml sterile tubes and stored at −80°C for 16S rRNA sequencing and VFA content determination.

**TABLE 1:** Diet ingredients and nutrition levels.

Item	Dietary energy, MJ/kg		
	
	MEA, 9.17	MEB, 10.00	MEC, 10.82
Ingredient, %			
Corn silage	40	25	10
Peanut seedling	30	30	30
Corn	5.44	22.25	39.06
Wheat bran	6.96	6.06	5.16
Soybean meal	14.60	13.69	12.78
Premix^1^	3	3	3
Total	100	100	100
Nutrient levels^2^			
Metabolic energy, MJ/kg	9.17	10.00	10.82
Crude fat, g/kg	20	22	23
Neutral-detergent fiber, g/kg	453	399	345
Acid-detergent fiber, g/kg	332	280	229
Crude ash, g/kg	67	61	54
Acid insoluble ash, g/kg	14	11	9
Crude protein, g/kg	132	130	129

*^1^Premix provides the following per kg: vitamin A 120 KIU; vitamin D_3_ 60 KIU; vitamin E 200 mg; Cu 0.15 g; Fe 1 g; Zn 1 g; Mn 0.5 g; I 15 mg; Se 5 mg; Co 2.5 mg; Ca 20 g; NaCl 100–250 g; and P 10 g.*

*^2^Except for metabolic energy, which is a predicted value, the rest are measured values.*

### Rumen Fermentation Parameters

The concentrations of total VFA (TVFA) and individual VFA were detected by gas chromatography (Agilent 7890A, NYSE: A, Palo Alto, CL, United States), referring to the method of Wang et al. (2020b). A total of 0.15 ml of 25% metaphosphoric acid was added into 1.5 ml of rumen fluid samples and was properly mixed. Subsequently, the mixed samples were stored at −20°C for more than 24 h and then centrifuged at 15,000 × *g* for 10 min at 4°C to take 1.0 ml of supernatant to filter with a 0.45-μm membrane. Then, filter liquor was injected into a special gas-phase vial, in which 1 μl of the sample was injected into an Agilent DB-FFAP gas-phase capillary column (30 m × 0.25 mm × 0.25 μm) automatically. The injector and detector temperatures were respectively set at 250 and 280°C, with the split ratio set at 50:1. The column was heated from 60 to 220°C at a rate of 20°C/min.

### Rumen and Intestinal Morphology

Hematoxylin–eosin (HE) staining and optical microscopy were used for the rumen and intestinal morphology analysis. According to the method described by Wang et al. (2020b) and Deng et al. (2020), paraffin sections of tissues were fixed. Formalin-immobilized duodenum, jejunum, ileum, and rumen samples were dehydrated and embedded in paraffin, subsequently embedded paraffin tissue was cut into cross sections of 5-μm thickness, and then the slices were stained with HE. The morphological structures of villus height (VH), villus width (VW), crypt depth (CD), and papillary height (PH) were acquired by using a microscope and an image processing and analysis system (Version 1, Leica Imaging Systems Ltd., Cambridge, United Kingdom). Using Image-Pro Plus 6.0 software, no fewer than 10 well-oriented intact villi and their corresponding crypts for each sample were measured; meanwhile, calculate the VH-to-CD ratio (VH/CD).

### DNA Extraction and PCR Amplification

Total genomic DNA from the rumen fluid samples was extracted by the CTAB/SDS method. The concentration and purity of DNA were monitored with 1% agarose gels and then using sterile water diluted to 1 ng/μl. The V3–V4 regions of distinct 16S rRNA genes (16S) were amplified with specific primers V515F (5′-GTGYCAGCMGCCGCGGTA A-3′) and V806R (5′-GGACTACHVGGGTWTCTAAT-3′). PCR amplification reactions were carried out in 30-μl systems with 15 μl of Phusion^®^ High-Fidelity PCR Master Mix (New England Biolabs), 10 ng of template DNA, and 0.2 μM of forward and reverse primers. PCR-amplified procedure initial denaturation was set at 98°C for 1 min, followed by 30 cycles of denaturation at 98°C for 10 s. Annealing was at 50°C for 30 s, and elongation was at 72°C for 30 s and finally at 72°C for 5 min. The amplified products were mixed with the same volume of 1× loading buffer (contained SYB green) and detected by 2% agarose gel electrophoresis. Then, using the GeneJET^TM^ Gel Extraction Kit (Thermo Scientific) to purify the mixture, PCR products were generated with the Ion Plus Fragment Library Kit 48 rxns (Thermo Scientific) to construct the sequencing libraries. The library quality was assessed on the Qubit@ 2.0 Fluorometer (Thermo Scientific). Finally, the Ion S5^TM^ XL platform was used to sequence the library and generate 407- to 412-bp single-end reads.

### Sequencing Analyses

Under specific filtering conditions, the raw reads were performed according to the Cutadapt quality control process to obtain high-quality clean reads (Martin, 2011). The UCHIME algorithm (Edgar et al., 2011) was used to detect chimera sequences and remove the chimera sequences (Haas et al., 2011) to finally obtain the clean reads compared to the SILVA database (the reference database) (Quast et al., 2012). Sequence analysis was implemented by Uparse Software (Uparse v7.0.1001) (Edgar, 2013), and sequences with ≥97% similarity were assigned to the same operational taxonomic units (OTUs). The SILVA database was used to annotate taxonomic information for each representative sequence screened for each OTU; meanwhile, a standard of sequence number corresponding to the sample with the least sequences was used to normalize the OTUs’ abundant information. Based on the output of normalized data, alpha diversity and beta diversity were analyzed subsequently. Alpha diversity was utilized to analyze the complexity of species diversity for each sample, including Shannon, Simpson, Chao1, ACE, Good-coverage, and Observed-species indices, and which were calculated by QIIME (Version1.7.0) and displayed by R Software (Version 2.15.3). Beta diversity is implemented in the evaluation of the differences in samples of species complexity. The UniFrac distance was calculated and the unweighted pair-group method with arithmetic means (UPGMA) sample cluster tree was constructed by QIIME software (Version 1.7.0). The principal coordinate analysis (PCoA) was performed to obtain principal coordinates and visualizations from complex, multidimensional data and display with the WGCNA package, stat packages, and ggplot2 package in R software (Version 2.15.3); the Vegan software package of R software was used for non-metric multi-dimensional scaling (NMDS) analysis. UPGMA clustering was performed to interpret the distance matrix using average linkage and was conducted by QIIME software. The R software was used to analyze the differences between groups of beta diversity index.

### Bacterial Metabolic Pathway and Function Predictions

Phylogenetic Investigation of Communities by Reconstruction of Unobserved States (PICRUSt) is based on the genetic information on the OTU and the Greengene database^1^ to construct the archaeal and bacterial domain and predict the full spectrum of the gene function. This is achieved by extrapolating the gene function spectrum of their common ancestors and other untested species in the Greengene database. Finally, the sequence of the microbial composition map is imported into the database to predict the metabolic function of microbiota. Furthermore, PICRUSt2^2^ was used to predict the metabolic function of rumen microorganisms; PICRUSt2 predictions based on the Integrated Microbial Genomes (IMG) database, including the Kyoto Encyclopedia of Genes and Genomes (KEGG) orthologs (KO) and Enzyme Commission numbers (EC numbers) and other several gene family databases, are supported by IMG (Douglas et al., 2020).

### Statistical Analysis

Preliminary experimental data were processed by Excel (Microsoft, Seattle, Washington, DC, United States), and SPSS 22 software (SPSS, Chicago, IL, United States) was performed for further statistical analysis. One-way ANOVA and *t*-tests were used to test the significance of rumen fermentation parameters and morphological indices. The microbial diversity, bacterial relative abundance, and function prediction analyses were carried out by the non-parametric test. The final results were expressed as means ± SEM. A *p*-value of <0.10 shows a trend, a *p*-value of <0.05 is significant, and *p* < 0.01 is extremely significant.

## Results

### Rumen Fermentation Parameters

The results of VFAs in the rumen are shown in Table 2. There was no significant difference linearly in the concentrations of acetate, propionate, butyrate, iso-butyrate, iso-valerate, valerate, and TVFA, and the ratio of acetate/propionate (Ac/Pr) in the rumen of fattening male Hu lambs with the increase of dietary energy level (one-way ANOVA and *t*-tests *p* > 0.05). The molar concentration of iso-valerate in the MEB group was significantly higher than the other two groups (quadratic, one-way ANOVA and *t*-tests *p* = 0.041). The molar proportion of iso-valerate in the MEB group was significantly higher than the other two groups (quadratic, one-way ANOVA and *t*-tests *p* = 0.033), while the molar proportion of acetate in the MEB group was dramatically lower than other two groups (quadratic, one-way ANOVA and *t*-tests *p* = 0.044) (Table 2). Actually, our previous research has found that the daily weight gain (ADG) and dry matter intake (DMI) are significantly increased, and feed conversion ratio (FCR) is dramatically decreased by increasing the dietary ME level in growth performance (Wang et al., 2020a).

**TABLE 2:** Effects of dietary energy levels on rumen fermentation parameters.

Item	Groups			SEM^2^	*p*-Value	
			
	MEA	MEB	MEC		Linear	Quadratic
Molar concentration (mM)						
Acetate	39.49	35.04	35.35	1.68	0.369	0.517
Propionate	9.59	8.92	8.47	0.49	0.419	0.922
Butyrate	4.84	4.34	4.30	0.23	0.402	0.654
Iso-butyrate	1.59	1.82	1.65	0.06	0.717	0.127
Iso-valerate	2.47^b^	2.98^a^	2.52^b^	0.11	0.865	0.041
Valerate	0.79	0.81	0.79	0.03	0.967	0.735
TVFA^1^	58.77	53.91	53.08	2.40	0.408	0.773
Ac/Pr	4.10	4.02	4.23	0.11	0.671	0.571
Molar proportion (mol/100 mol)						
Acetate	66.99^a^	65.05^b^	66.70^a^	0.46	0.695	0.044
Propionate	16.36	16.38	15.86	0.36	0.574	0.878
Butyrate	8.16	8.13	8.06	0.16	0.765	0.877
Iso-butyrate	2.78	3.38	3.13	0.12	0.229	0.091
Iso-valerate	4.34^b^	5.54^a^	4.77^b^	0.23	0.389	0.033
Valerate	1.36	1.52	1.48	0.04	0.298	0.348

*Values within a row with different superscripts (a,b) differ significantly at *p* < 0.05.*

*^1^Total volatile fatty acids. ^2^SEM, standard error of the mean.*

### Rumen and Intestinal Morphology

**FIGURE 1:**
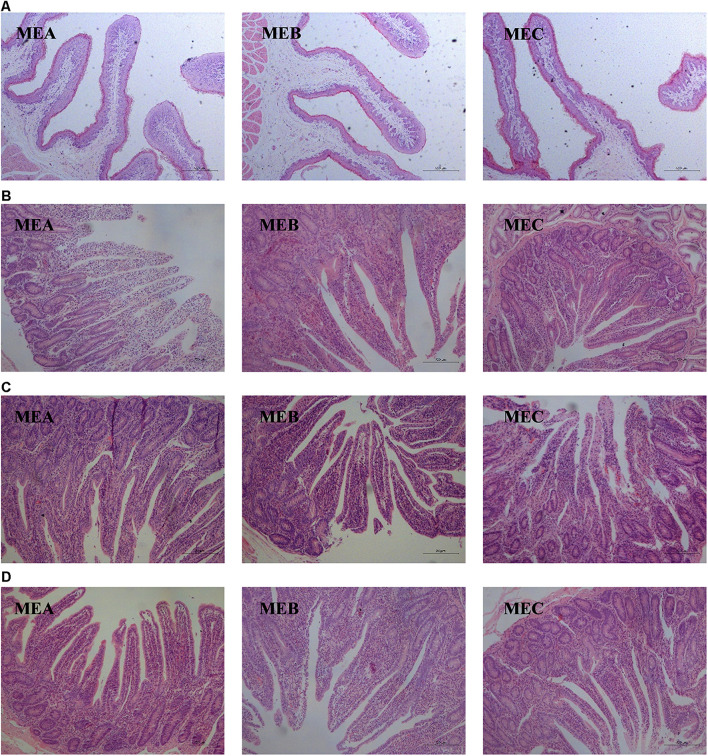
Effects of low-, medium-, and high-energy diets on rumen and small intestinal morphology of male Hu lambs. **(A)** Papillary height of rumen. Scale bar, 500 μm. **(B)** Duodenum. Scale bar, 200 μm. **(C)** Jejunum. Scale bar, 20 μm. **(D)** Ileum. Scale bar, 200 μm.

The rumen and intestinal morphology are shown in Table 3 and Figure 1. By increasing the dietary energy level, the CD (linear, one-way ANOVA and *t*-tests *p* = 0.000 and quadratic, one-way ANOVA and *t*-tests *p* = 0.002) was dramatically increased, while the VH/CD was significantly decreased (linear, one-way ANOVA and *t*-tests *p* = 0.002) in the duodenum. In the jejunum, the CD (linear, one-way ANOVA and *t*-tests *p* = 0.012 and quadratic, one-way ANOVA and *t*-tests *p* = 0.033) was also significantly increased. There were significant differences in the VH (linear, one-way ANOVA and *t*-tests *p* = 0.000 and quadratic, one-way ANOVA and *t*-tests *p* = 0.018), VW (linear, one-way ANOVA and *t*-tests *p* = 0.004 and quadratic, one-way ANOVA and *t*-tests *p* = 0.002), and VH/CD (linear, one-way ANOVA and *t*-tests *p* = 0.005) in the ileum; the VH and VH/CD increased as the diet energy level increases, while the VW in the MEA group was significantly higher than the other two groups. The rumen PH was the highest in the MEB (medium energy) group (quadratic, one-way ANOVA and *t*-tests *p* = 0.043).

**TABLE 3:** Effects of dietary energy levels on GIT morphology.

Item	Measured index (μm)	Groups			SEM	*p*-Value	
				
		MEA	MEB	MEC		Linear	Quadratic
Duodenum	Villus height	362.12	390.08	354.57	12.75	0.825	0.264
	Crypt depth	156.53^b^	239.48^a^	229.13^*a*^	11.66	0.000	0.002
	Villus width	127.34	133.44	123.79	2.86	0.636	0.210
	VH/CD	2.34^a^	1.64^b^	1.55^b^	0.12	0.002	0.072
Jejunum	Villus height	363.75	338.43	428.97	19.00	0.125	0.165
	Crypt depth	184.94^b^	163.8^b^	254.59^a^	14.86	0.012	0.033
	Villus width	131.76	129.64	141.25	2.83	0.152	0.285
	VH/CD	1.99	2.10	1.70	0.08	0.079	0.113
Ileum	Villus height	355.73^b^	362.09^b^	463.45^a^	15.12	0.000	0.018
	Crypt depth	195.12	175.59	177.05	8.91	0.455	0.600
	Villus width	156.83^a^	137.13^b^	142.67^b^	2.76	0.004	0.002
	VH/CD	1.86^b^	2.12^b^	2.66^a^	0.12	0.005	0.470
Rumen	Papillary height	1,648.41^b^	2,000.15^a^	1,706.07^b^	70.76	0.693	0.043

*Values within a row with different superscripts (a,b) differ significantly at p < 0.05.*

### Sequences Across Different Diet Groups

Sequencing results showed that the average of 84,736 reads for each sample was measured through shearing and filtration, and an average of 80,128 valid data reads was obtained through quality control, and the quality control efficiency was 94.61%. A total of 2,456 OTUs were acquired for all samples. Among them, 2,453 (99.88%) OTUs were annotated at the kingdom level, 2,365 (96.29%) at the phylum level, 2,310 (94.06%) at the phylum level, 2,171 (88.40%) at the order level, 1,856 (75.57%) at the family level, 603 (24.55%) at the genus level, and 197 (8.02%) OTUs annotated at the species level (Supplementary Table 1). The OTU number, which reflects the rumen microbial diversity, was significantly (non-parametric test *p* = 0.013) decreased by increasing the dietary energy level. The richness estimator indices of ACE (non-parametric test *p* = 0.085) and Chao1 (non-parametric test *p* = 0.063) showed a downward trend as dietary energy level increased, whereas there were no significant differences in the alpha diversity indices of Shannon and Simpson and the phylogenetic diversity index of PD-whole-tree (non-parametric test *p* > 0.05). Additionally, the goods-coverage index was greater than 99%, indicating that the sequencing depth basically covered all the species in all the samples and accurately reflected the microbial community (Table 4). The rarefaction curves tended to be flat at 3% divergence and also showed that the sequencing data were reasonable and that the sequencing depth sufficiently covered the majority of bacterial diversity in all samples (Supplementary Figure 1).

**TABLE 4:** Rumen microbiota diversity indices of different dietary energy levels.

Item	Groups			SEM^6^	*p*-Value
		
	MEA	MEB	MEC		
OTUs^1^	1,514.60^a^	1,450.20^ab^	1,365.40^b^	26.32	0.013
Shannon indices^2^	8.27	8.11	8.11	0.08	0.932
Simpson indices^2^	0.99	0.99	0.99	0.00	0.704
ACE value^3^	1,606.07	1,532.01	1,467.62	27.83	0.085
Chao1 value^3^	1,605.26	1,530.87	1,578.59	51.36	0.063
Goods-coverage^4^	0.997	0.997	0.997	0.00	0.985
PD-whole-tree^5^	103.82	102.26	94.84	2.09	0.134

*Values within a row with different superscripts (a,b) differ significantly at *p* < 0.05. OTU, operational taxonomic units; ACE, abundance-based coverage estimator.*

*^1^Number of operational taxonomic units. ^2^Shannon and Simpson indices. ^3^ACE and Chao species richness estimators. ^4^Sequencing depth index. ^5^Phylogenetic diversity index. ^6^SEM, standard error of the mean.*

### Rumen Microbial Diversity

**FIGURE 2:**
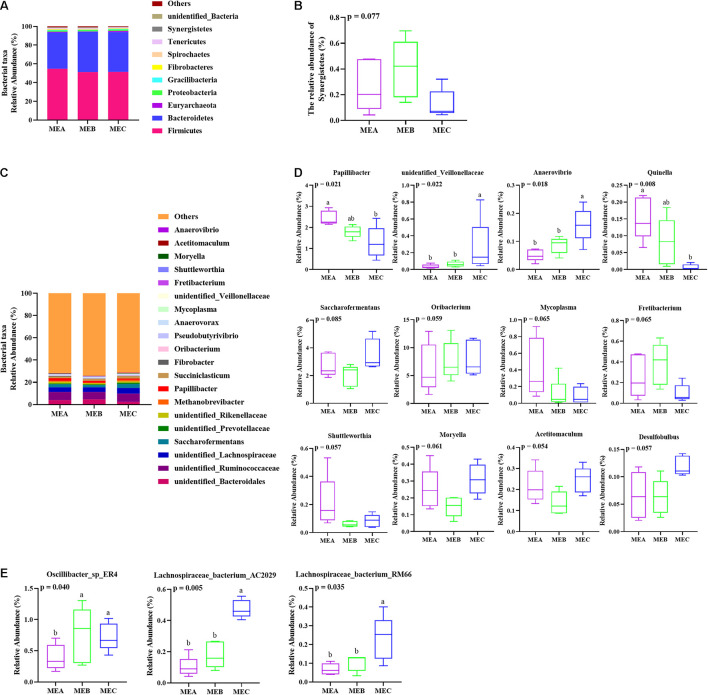
Effects of low-, medium-, and high-energy diets on rumen microbiota in male Hu lambs. **(A)** Bacterial taxonomic profile of the rumen microbiota at the phylum level. **(B)** Relative abundances of *Synergistetes*. **(C)** Relative abundances of the dominant genera in the rumen. **(D)** Relative abundance of representative genera. **(E)** Relative abundance of representative species. Values with different superscripts (a,b) differ significantly at *p* < 0.05.

The taxonomic analysis results showed that 24 phyla were detected and that unclassified bacteria were excluded. The five phyla with the highest relative abundance were *Firmicutes*, *Bacteroidetes*, *Euryarchaeota*, *Proteobacteria*, and *Gracilibacteria* (Figure 2A). As expected, the relative abundance of *Firmicutes*, *Bacteroidetes*, and *Euryarchaeota* was the richest in the three experimental groups, accounting for about 95% of the total abundance. The relative abundance of *Synergistetes* first increased and then decreased (non-parametric test *p* = 0.077) with the increase in dietary energy levels (Figure 2B).

At the genus level, 124 genera were identified and the 10 most relatively abundant genera were elucidated in all the samples (Supplementary Table 2 and Figure 2C). Within three groups, the five dominant relative genera were *unidentified Ruminococcaceae*, *unidentified Lachnospiraceae*, *unidentified Bacteroidales*, *Saccharofermentans*, and *Papillibacter*. Among the rest, *unidentified Ruminococcaceae* and *unidentified Lachnospiraceae* were members of *Firmicutes* in the phylum, *unidentified Bacteroidales* is from *Bacteroidetes*, and *Saccharofermentan*s and *Papillibacter* belonged to *Clostridiales* of *Firmicutes*.

The relative abundance of *Papillibacter* (non-parametric test *p* = 0.021) and *Quinella* (non-parametric test *p* = 0.008) was significantly decreased by increasing dietary energy, while the relative abundance of *unidentified_Veillonellaceae* (non-parametric test *p* = 0.022) and *Anaerovibrio* (non-parametric test *p* = 0.018) increased significantly with the increase in dietary energy level (Figure 2D). The relative abundance of *Oribacterium* showed an upward tendency (non-parametric test *p* = 0.059), the relative abundance of *Mycoplasma* presented a downward trend (non-parametric test *p* = 0.065), the relative abundance of *Fretibacterium* showed a tendency of rising after falling (non-parametric test *p* = 0.065), and the relative abundance of *Saccharofermentans* (non-parametric test *p* = 0.085), *Shuttleworthia* (non-parametric test *p* = 0.057), *Moryella* (non-parametric test *p* = 0.061), *Acetitomaculum* (non-parametric test *p* = 0.054), and *Desulfobulbus* (non-parametric test *p* = 0.057) first decreased and then increased with the increase in dietary energy levels (Figure 2D). Additionally, the relative abundance of *Oscillibacter*_sp_ER4 (non-parametric test *p* = 0.040) in both MEB and MEC groups was significantly higher than the MEA group, the relative abundance of *Lachnospiraceae*_bacterium_AC2029 (non-parametric test *p* = 0.005) and *Lachnospiraceae_bacterium*_RM66 (non-parametric test *p* = 0.035) in the MEC group was significantly higher than that in MEA and MEB groups at the species level (Figure 2E), and the three kinds of bacteria all belonged to *Firmicutes*.

### Rumen Bacteria Clustering Dissimilarities

**FIGURE 3:**
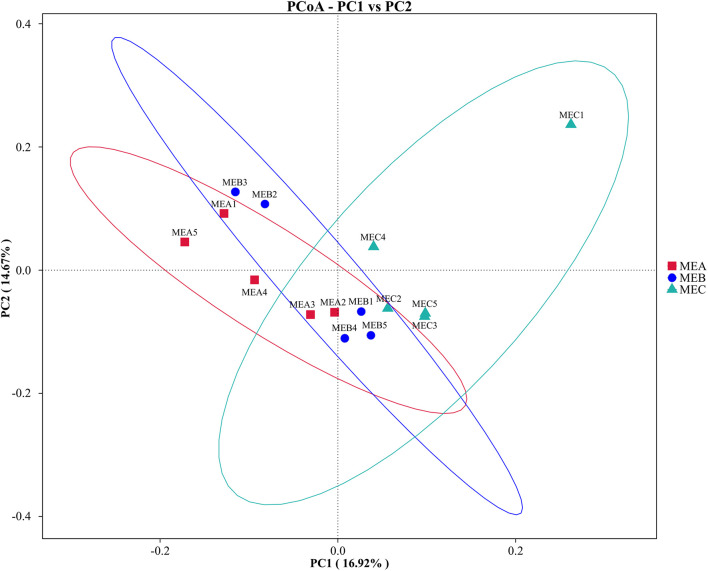
Principal coordinate analysis (PCoA) of low-, medium-, and high-energy diets based on unweighted UniFrac distances. MEA1 to MEA5 in group MEA (red); MEB1 to MEB5 in group MEB (blue); and MEC1 to MEC5 in group MEC (green). PC, percent variation explained by the axis.

The PCoA showed that the rumen microbial diversity accounted for 16.92% variation in the MEC group, distinguished from both MEA and MEB groups by PC1, and that the microbial diversity between MEA and MEB groups represented 14.67% variation of PC2 (Figure 3). The NMDS analysis also verified that the rumen bacterial diversity in the MEC group was clustered discretely with the other two groups (Stress = 0.098), which could accurately reflect the degree of variation for all samples (Supplementary Figure 2).

### Rumen Microbial Function Prediction

**FIGURE 4:**
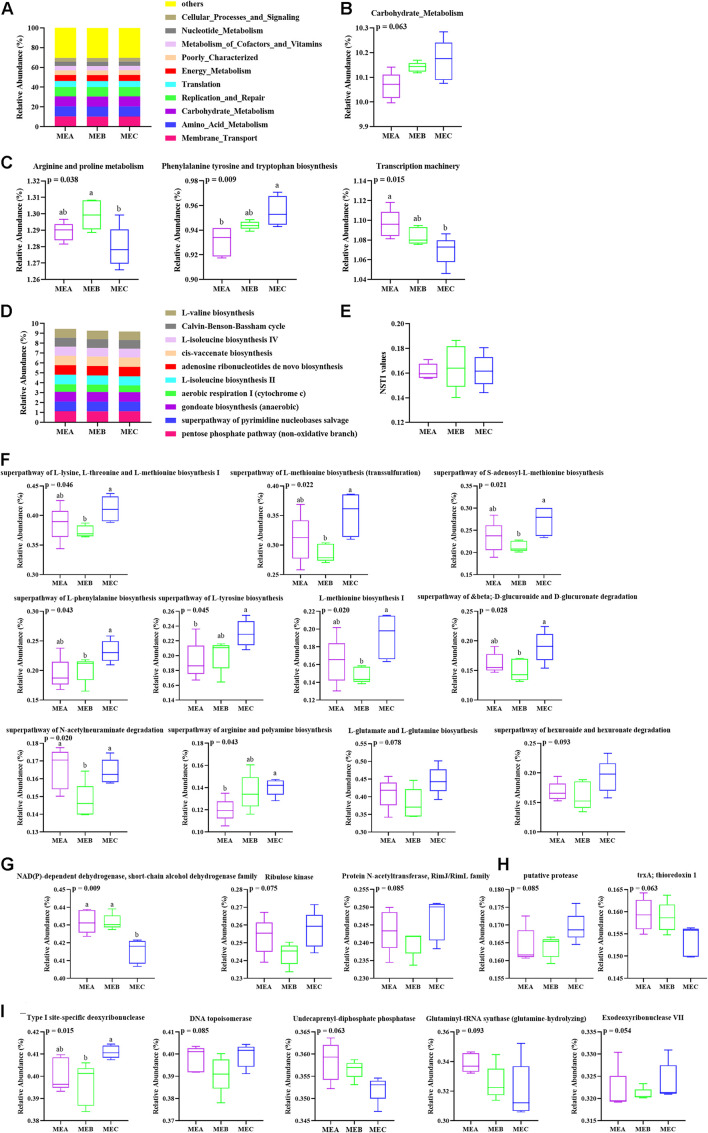
Functional prediction of low, medium, and high dietary energy of rumen microbial community and related pathways. **(A)** Relative abundance of the dominant pathways annotated to KEGG level 2. **(B)** Relative abundance of carbohydrate metabolism. **(C)** Relative abundance of representative pathways at KEGG level 3. **(D)** The top 10 relative abundance of KEGG pathways by PICRUSt2. **(E)** The NSTI values. **(F)** The differential metabolic pathways by PICRUSt2. **(G)** The variation in top 35 relative abundances of COG analysis. **(H)** The variation in top 35 relative abundances of KO analysis. **(I)** The variation in top 35 relative abundances of EC analysis. Values with different superscripts (a,b) differ significantly at *p* < 0.05.

“Metabolism” with more than 47% of the total reads has the highest relative abundance among the three groups at KEGG level 1 (Supplementary Figure 3). The 35 gene families of the most relative abundant (relative abundance >0.08%) from each rumen sample were present at KEGG level 2 (Supplementary Table 3). The genes related to membrane transport, amino acid metabolism, carbohydrate metabolism, replication and repair, and energy metabolism exhibited the most relative abundance among the three groups (Figure 4A), in which carbohydrate metabolism showed an obviously increased trend (non-parametric test *p* = 0.063) by increasing the dietary energy level (Figure 4B). At KEGG level 3, the pathways referring to transporters, general function prediction only, DNA repair and recombination proteins, ATP-binding cassette (ABC) transporter and ribosome, purine metabolism, and pyrimidine metabolism were highly represented (Supplementary Table 4). With an increase in the dietary energy level, three pathways showed significant variation; the relative abundance of phenylalanine tyrosine and tryptophan biosynthesis (non-parametric test *p* = 0.009) had notably increased, the relative abundance of arginine and proline metabolism significantly increased after falling (non-parametric test *p* = 0.038), whereas the abundance of the transcription machinery (non-parametric test *p* = 0.015) pathway had dramatically decreased (Figure 4C). Results of rumen microbial function prediction by PICRUSt2 are shown in Figures 4D–I. The top 10 relative abundance pathways are shown in Figure 4D, but there was no significant difference among the three groups, including the top 35 relative abundance pathways, whereas there were nine predictive metabolic pathways that were significantly affected by dietary energy levels and two other pathways showed a certain trend (Figure 4F). In addition, the NSTI (Nearest Sequenced Taxon Index) values are evaluated in Figure 4E, and the NSTI values were lower than 0.18 among different groups. The results of COG (Clusters of Orthologous Groups) functional annotation analysis showed that the relative abundance of NAD(P)-dependent dehydrogenase, short-chain alcohol dehydrogenase family was significantly higher (non-parametric test *p* = 0.009) in MEA and MEB than in MEC (Figure 4G). The results showed that the top 35 relative abundance based on KO analysis had no significant difference among the three groups (Figure 4H). The EC analysis results showed that the relative abundance of Type I site-specific deoxyribonuclease (non-parametric test *p* = 0.015) was significantly affected by different dietary energy levels (Figure 4I).

## Discussion

The rumen is a unique digestive organ, which is known to be the most powerful natural fermentation tank to degrade fiber materials, and plays an extremely crucial role in nutrient digestion and metabolism in ruminants. The complex microbiota in the rumen played a critical role in feed fermentation and energy metabolism (Flint et al., 2007). The fermentation and degradation of feed in the rumen are closely related to the composition of rumen microbiota. About 70% of the energy required by rumen fermentation is provided by VFAs to ensure the maintenance, growth, and production performance of the host. Therefore, the rumen microbial composition and structure affected the nutritional health and growth of ruminants (Pokharel et al., 2018). On the contrary, the host provided fermentation substrates and a suitable anaerobic environment for the survival of rumen microorganisms (Guan et al., 2008). In the present study, the experimental population was assigned to three groups according to the dietary energy level: low (MEA), medium (MEB), and high (MEC) energy.

Previous studies have demonstrated that the feed type and nutrient level can influence the proportion of acetate, propionate, and butyrate; in particular, a concentrate-based or a high-energy diet is capable of increasing the proportion of propionate in the rumen (van Soest, 1994; Keady et al., 2001; Agle et al., 2010). In the present study, different dietary energy levels had no significant effect on the molar concentration of acetate, propionate, butyrate, and TVFA, and these results were in agreement with other studies (Murphy et al., 2000). Interestingly, the medium energy level significantly increased the molar concentration and proportion of iso-valerate at the quadratic level, while quadratically decreasing the proportion of acetate compared to the low- and high-energy groups; these results were not completely consistent with previous studies (Shabat et al., 2016), whether these findings had beneficial effects on the composition and function of rumen microbiota remained to be explored. Unexpectedly, the concentrations of acetate, propionate, butyrate, and TVFA were lower in the medium- and high-energy groups compared with the low-energy group in the present study, which might be related to the increase of high corn content in the medium- and high-energy diet and excessive fasting times during sampling (more than 12 h). Guan et al. (2008) and Ellison et al. (2017) found that the feed composition and rumen environment could affect the feed conversion efficiency in ruminants, and high-grain diets might be easier to digest and absorb. Accordingly, the concentration of VFAs in rumen fluid would decrease faster. Thus, it can be seen that iso-valerate and acetate responded to alter dietary energy levels.

The primary site of nutrient digestion and absorption is the GIT of ruminants. Steele et al. (2016) and Wester et al. (1995) found that the dietary energy density and ME intake can affect the GIT tissues, such as the height and width of rumen papillae, which are generally used to estimate the rumen epithelium growth (Steele et al., 2014). Khan et al. (2011) found that the particle size and diet composition can affect the morphological structure of rumen papillae. A previous study has also shown that the high-energy diet could stimulate the proliferation of rumen papillae of goats (Shen et al., 2004). Papillae height was the highest in the medium-energy group compared with the low- and high-energy groups in this study, which suggested that an appropriate increase in dietary energy level might contribute to the growth and development of rumen papilla. This result was consistent with that of a previous experiment (Kim et al., 2012).

Villus height, VW, CD, and VH/CD are acknowledged as key histomorphology figures to reflect the digestive and absorption functions of the small intestine (Hedemann et al., 2003; Yang et al., 2013, 2016). Intestinal epithelial cell proliferation has a positive impact on VH and CD. Wang et al. (2019) found that VH was positively correlated with the absorption capacity of nutrients and CD had a synergistic effect on villus cell renewal. In the present study, the VH of ileum significantly increased in the high-energy group, and the CD of the duodenum and jejunum significantly increased in the medium- and high-energy groups, while a significant decrease was observed in the VH/CD of the duodenum and the VW of the ileum in the medium- and high-energy groups. Previous studies have found that nutritional restriction in weaned lambs could reduce the CD of jejunum (Lingyan et al., 2014), while supplementary concentration could promote the VH of grazing calves (Azim et al., 2011), and our study showed similar results. Wang et al. (2009) and Mcleod and Baldwin (2000) found that increasing dietary ME could strengthen Na^+^-K^+^-ATPase activity in the small intestine, which contributed to promote the proliferation of intestinal epithelial cells and VH formation of ruminants. These results demonstrated that dietary energy levels might lead to a dynamic effect on the intestinal morphology of male Hu lambs, and there are many similarities between our results and those of previous studies; the high-energy group was recommended as an appropriate dietary energy level to promote the intestinal development and improve intestinal digestion and absorption function.

The correlation between dietary energy levels and rumen microbiota in fattening male Hu lambs was investigated by using an Ion S5TMXL sequencing platform to sequence the V3–V4 regions of the rumen microbial 16S rDNA gene. The alpha diversity index mainly reflects the richness and evenness of the species in the samples. A previous study has shown that a high-grain diet could notably decrease the OTUs number (Zhang et al., 2017). The present study also revealed that OTUs significantly decreased as the dietary energy level increased, which implies that the high energy level (or a concentrated level of diet) might affect rumen microbial diversity and the relative abundance of some microorganisms. The chao1 and ACE values observed in the low energy level were higher than those in the medium and high energy levels. Our results indicated that the relative abundance of rumen microbial communities was influenced by the different energy levels in the diets, and the rumen microecological environment was relatively stable and microbial communities were rich and diverse in low and medium energy level, which might be conducive to rumen health and the absorption and transformation of nutrients in ruminants (Liu et al., 2013).

From the composition of the rumen microorganisms, the types and proportions of the dominant microbiota in the rumen of lambs were similar among the three groups (low, medium, and high energy level), indicating that the rumen microbial communities of male Hu lambs in the fattening stage were relatively stable, and dietary energy levels had no significant impact on the dominant microbial community in rumen. At the phylum level, the three dominant microbial phyla were *Firmicutes*, *Bacteroidetes*, and *Proteobacteria* in three experimental groups, and this result was consistent with previous research findings in goats (Ye et al., 2017; Zhang et al., 2017) and cattle (Plaizier et al., 2017; Wetzels et al., 2017). The relative abundance of the dominant genera also did not change significantly at the genus level, which was in agreement with the results of Wang et al. (2016). Notably, the present study revealed large numbers of bacteria related to unclassified and uncultured genera in the rumen of Hu sheep. Wang Y. et al. (2017) found a similar pattern in Tan sheep. This result suggested that sheep might have a more diverse rumen microbiome and exhibit great differences among different breeds. Hook et al. (2011) and Wang Y. et al. (2017) also have reported that *Firmicutes* and *Bacteroidetes* were the dominant phyla in rumen, which were closely associated with carbohydrate and protein metabolism. *Firmicutes* and *Bacteroidetes* were also the most dominant phyla in the current study, and our results further verified that dietary energy level had no significant effect on the dominant rumen microbiota in Hu sheep. Additionally, the different dietary treatments can affect the ratios of *Firmicutes* and *Bacteroidetes*, which might obstruct the rumen bacterial function. Pitta et al. (2016) found that *Bacteroidetes*, compared to *Proteobacteria*, has a stronger ability to degrade carbohydrates and can efficiently degrade polysaccharides and protein (Huo et al., 2014). Previous studies also reported that dietary nutrient level could influence the rumen bacterial composition of sheep (Wang Y. et al., 2017; Seddik et al., 2019). Golder et al. (2014) and Metzler-Zebeli et al. (2015) have shown that the relative abundance of *Proteobacteria* was the third highest at the phylum level, unexpectedly much lower than *Firmicutes* and *Bacteroidetes*, and the present study obtained the same consistent result as above. However, Pitta et al. (2016) found that *Proteobacteria* plays an important role in rumen metabolism, such as insoluble sugar digestion and biofilm formation. Besides, in the current study, *Fibrobacteres* was less abundant at the phylum level, but this observation was confirmed by the previous result of Pitta et al. (2014).

At the genus level, the variations of the rumen microbiome population within the represented genera were identified among three groups with different dietary energy levels in the present study, such as *unidentified Ruminococcaceae* comprising 6.68–7.36% of the total bacteria, which is clearly inconsistent with previous studies (Wang Y. et al., 2017; Seddik et al., 2019). Additionally, breed, age, feeding, management, season, herding, and geographic regions may also influence the characteristics of rumen microbial communities in ruminants (de Menezes et al., 2011). Recent research has shown that *Ruminococcaceae* bacteria played a critical role in dietary energy and lipid metabolism. Menni et al. (2018) found that diversity was negatively correlated with vascular hardness. The present study indicated that *unidentified Ruminococcaceae* was the most relatively abundant genus in all three treatments. *Papillibacter* is a butyrate-producing bacterium; restricted feeding could increase its abundance in sheep (Hu et al., 2018), whose relative abundance was markedly decreased and the butyrate concentration was decreased with increasing dietary energy. The relative abundance of propionate producing *Quinella* also significantly decreased, and the concentration of propionate showed a corresponding decline by increasing dietary energy level. Previous research found that *Veillonellaceae* was positively associated with inflammatory bowel disease (Atarashi et al., 2017), while in the present study, the relative abundance of *unidentified_Veillonellaceae* in the high-energy group was dramatically higher than that in both low- and medium-energy groups. *Anaerovibrio* are typically fat decomposers, which can hydrolyze triglycerides to glycerol and fatty acids, and further convert glycerol to propionate and succinate (Ren et al., 2019); the relative abundance of *Anaerovibrio* was highest in the high-energy group in the present study. These results indicated that high dietary energy altered bacterial community and damaged the rumen development. Long-term feeding of high-energy diet might affect the gastrointestinal health of lambs and lead to excessive fat deposition. Furthermore, beta diversity analysis of PCoA and NMDS revealed the distinct bacterial compositions with three diets of different energy.

Functional prediction of rumen bacteria by PICRUSt showed that the number of metabolism-related pathways was enriched at KEGG level 2, in which the membrane transporter was the most relatively abundant; carbohydrate metabolism, energy metabolism, amino acid metabolism, replication and repair, and translation were all general metabolic functions and essential for survival, reproduction, and growth of GIT microorganisms (Lamendella et al., 2011). These results are consistent with previous studies (Wang Y. et al., 2017). In particular, the relative abundance of carbohydrate metabolism-related genes showed a clear upward trend as dietary energy increases. Gifford et al. (2013) found that ribosomes played a crucial role in protein synthesis, and Yan et al. (2016) showed that ABC transporters were closely related to nutrient uptake. In the current study, the pathways of both ABC transporter and ribosome were enriched and all the related genes showed high relative abundance in the three groups at KEGG level 3. The present study also showed that the genes responsible for phenylalanine, tyrosine, and tryptophan biosynthesis were up-regulated by increasing dietary energy, which indicated enhanced fermentation and metabolic activities of rumen microorganisms, whereas the decrease in the arginine and proline metabolism and transcription machinery-related genes could be indicators of the high-energy diet influencing the function of the rumen. Furthermore, the rumen microbial functions predicted by PICRUSt2 showed that the relative abundances of KEGG pathways were significantly affected by dietary energy levels, but the top 35 relative abundance pathways had no significant difference in the three groups. Results of COG analysis showed that only the relative abundance of NAD(P)-dependent dehydrogenase, a short-chain alcohol dehydrogenase family, was significantly affected by different dietary energy levels, such as in the high-energy group, but lower in the low- and medium-energy groups. NAD(P)-dependent dehydrogenase, a short-chain alcohol dehydrogenase, is known to play critical roles in amino acid, carbohydrate, and lipid metabolism (Kavanagh et al., 2008). There were no significant differences in the top 35 relative abundances based on KO functional analysis with different dietary energy levels, while the relative abundance of Type I site-specific deoxyribonuclease was significantly affected by low, medium, and high dietary energy levels. Additionally, the NSTI values did not exceed 0.18, indicating the prediction reliability of rumen microbial metabolism by PICRUSt2. These results were inconsistent with the results of previous studies (Seddik et al., 2019). It is also implied that increasing the dietary energy level could alter the rumen microbial diversity and also influenced their corresponding metabolic function.

## Conclusion

In conclusion, this study primarily investigated rumen fermentation and histological and microbial diversity in fattening male Hu lambs fed diets with different energy levels. The results suggested that the concentration and molar proportion of rumen iso-valerate were significantly higher in the medium-energy group; also, the molar proportion of iso-butyrate was higher in the medium-energy group than in both low- and high-energy groups. The rumen PH was significantly higher in the medium-energy group than in both low- and high-energy groups. In addition, the duodenum CD and VH/CD, jejunum CD, and ileum VH, VW, and VH/CD were dramatically affected by dietary ME levels; a high energy level might be better for digestion and absorption in the small intestine. The diversity index of OTUs was significantly lower in the high-energy group compared to the low- and medium-energy groups. The dietary energy level significantly affected the relative abundance of *Papillibacter*, *Quinella*, *unidentified_Veillonellaceae*, and *Anaerovibrio*, while the majority of rumen microbial community and corresponding functions were consistent and were not affected by dietary ME. Taking the above points into consideration, the medium-energy group (metabolizable energy of 10.00 MJ/kg) was recommended as the appropriate dietary energy level in intensive fattening of Hu lambs, and these findings provided important theory reference and production practice guidance.

## Data Availability Statement

The datasets presented in this study can be found in online repositories. The names of the repository/repositories and accession number(s) can be found below: https://www.ncbi.nlm.nih.gov/, SRR11829336–SRR11829350.

## Ethics Statement

The animal study was reviewed and approved by the Animal Care Committee of Hunan Normal University in reference to the Administration of Affairs Concerning Experimental Animals.

## Author Contributions

HY and QW: conceptualization. XW and YW: methodology. PH and XZ: software. MZ: validation. YZ: formal analysis. QW: investigation and writing—original draft preparation. CD: resources. JH: data curation. TH: writing—review and editing. MZ: visualization. JL: supervision. HY: project administration and funding acquisition. All authors have read and agreed to the published version of the manuscript.

## Conflict of Interest

QW and CD were employed by the company Hubei Zhiqinghe Agriculture and Animal Husbandry Co., Ltd. The remaining authors declare that the research was conducted in the absence of any commercial or financial relationships that could be construed as a potential conflict of interest.

## Publisher’s Note

All claims expressed in this article are solely those of the authors and do not necessarily represent those of their affiliated organizations, or those of the publisher, the editors and the reviewers. Any product that may be evaluated in this article, or claim that may be made by its manufacturer, is not guaranteed or endorsed by the publisher.

## Footnotes

1
https://picrust.github.io/picrust/

2
https://github.com/picrust/picrust2/wiki

## Supplementary Material

Supplementary Figure 1 Rarefaction analyses of the different samples. Rarefaction curves of all samples and OTUs clustered at 97% similarity.

Supplementary Figure 2 NMDS analysis results based on OTU level from different dietary energy levels.

Supplementary Figure 3 The relative abundance of different functional taxa at level 1, annotated based on the KEGG orthologs datasets at different dietary nutrient levels.

Supplementary Table 1 Overview of species annotation for all samples.

Supplementary Table 2 Variance analysis of taxonomic composition of the 10 most abundance in phylum level and genus level of rumen bacterial community fed different dietary energy levels.

Supplementary Table 3 Predicted functions at level 2 of the rumen bacterial microbiota of male Hu lambs fed different dietary energy levels.

Supplementary Table 4 The 35 most abundant KEGG pathways at level 3 in the rumen bacteria of male Hu lambs fed different dietary energy levels.
