# Supplementary material for: Effects of Dietary Energy Levels on Rumen Fermentation, Gastrointestinal Tract Histology, and Bacterial Community Diversity in Fattening Male Hu Lambs
Source: Front Microbiol. 2021 Sep 10;12:695445. doi: 10.3389/fmicb.2021.695445 (PMC8460862; doi:10.3389/fmicb.2021.695445)
Supplement: Supplementary Figure 1 — Rarefaction analyses of the different samples. Rarefaction curves of all samples and OTUs clustered at 97% similarity. [file Table_1.DOCX]

Supplementary Material

# Supplementary Figures and Tables

## Supplementary Figures


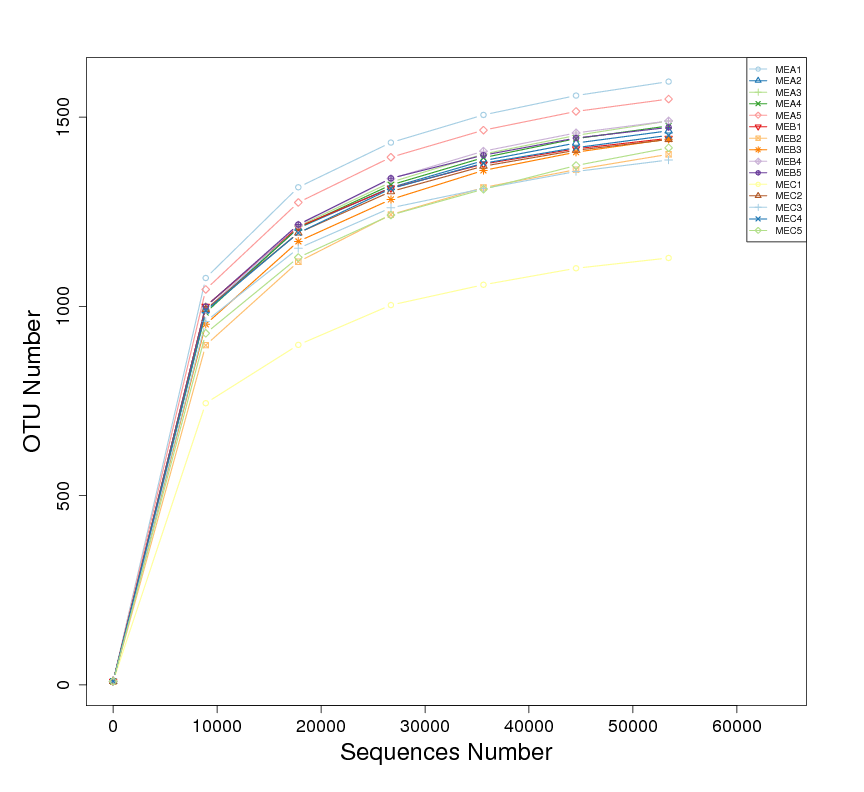


**Supplementary Figure 1.** Rarefaction analyses of the different samples. Rarefaction curves of all samples and OTUs clustered at 97% similarity. The samples of group MEA included sheep MEA1, MEA2, MEA3, MEA4 and, MEA5 which were fed the diet was 9.17MJ/kg of metabolizable energy; the group MEB samples included sheep MEB1, MEB2, MEB3, MEB4 and, MEB5, which were fed the diet was 10.00 MJ/kg of metabolizable energy; and the group MEC samples included sheep MEC1, MEC2, MEC3, MEC4 and, MEC5, which were fed the diet was 10.82 MJ/kg of metabolizable energy. The following analysis was also based on these conditions.


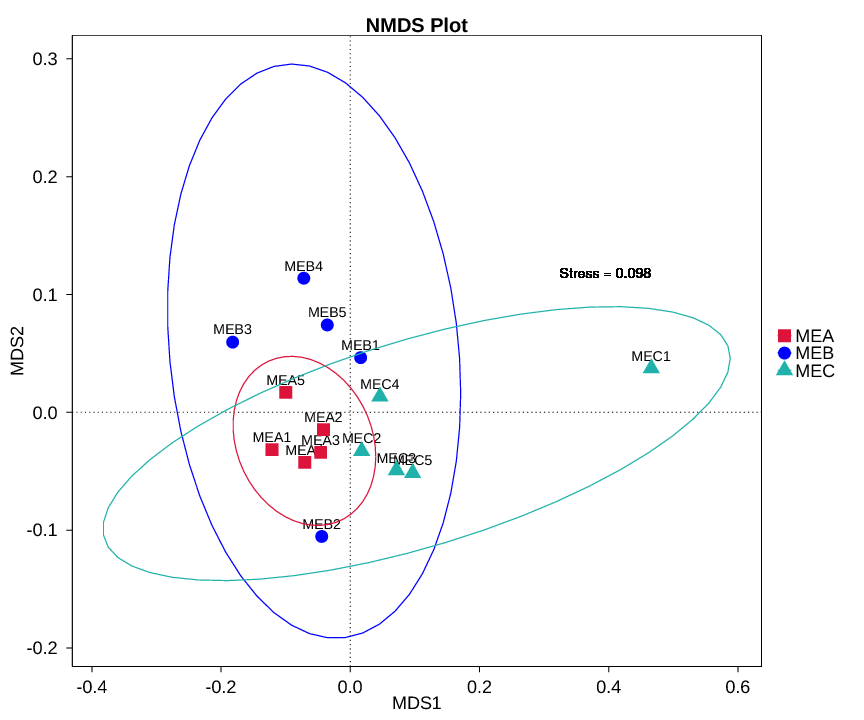


**Supplementary Figure 2.** NMDS analysis results based on OTU level from different dietary energy levels. Individual samples from MEA1 to MEA5 in group MEA (red); MEB1 to MEB5 in group MEB (blue); MEC1 to MEC5 in group MEC (green).


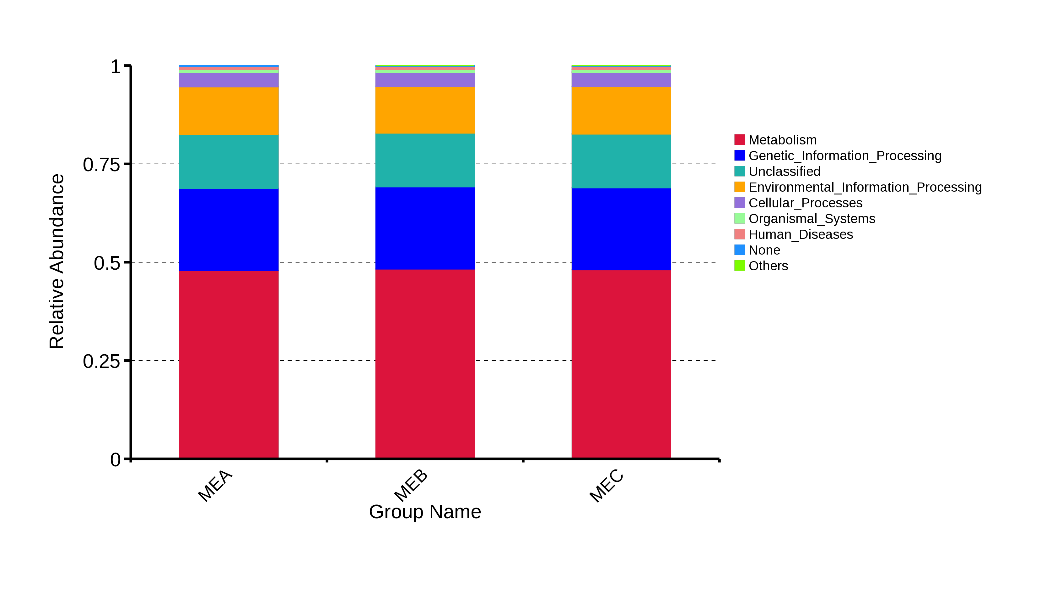


**Supplementary Figure 3.** The relative abundance of different functional taxa at level 1, annotated based on the KEGG Orthologs datasets at different dietary nutrient levels.

## Supplementary Tables

**Supplementary Table 1.** Overview of species annotation for all samples

| **Item** | **Number or proportion** | **Dominant species** |
| --- | --- | --- |
| OTU catalogue^1^ | 2,456 | - |
| Annotated on database | 2,453(99.88%) | - |
| Annotated on Unclassified | 3(0.12%) | - |
| Annotated on Kingdom level | 99.88% | - |
| Annotated on Phylum level | 96.29% | *Firmicutes, Bacteroidete, Euryarchaeota* |
| Annotated on Class level | 94.06% | *Clostridia, Bacteroidia, Negativicutes* |
| Annotated on Order level | 88.40% | *Clostridiales, Bacteroidales, Selenomonadales* |
| Annotated on Family level | 75.57% | *Ruminococcaceae, Christensenellaceae, Rikenellaceae* |
| Annotated on Genus level | 24.55% | *unidentified_Bacteroidales, unidentified_Ruminococcaceae, unidentified_Lachnospiraceae* |
| Annotated on Species level | 8.02% | *Bacteroidales_bacterium*_Bact_22, *rumen_bacterium*_NK4A214, *rumen_bacterium*_YS3 |

OTU = operational taxonomic unit.

^1^Total number of operational taxonomic units for all samples.

**Supplementary Table 2.** Variance analysis of taxonomic composition of the 10 most abundance in phylum level and genus level of rumen bacterial community fed different dietary energy levels

| **Item** | **Groups** | | | **SEM** | ***p-value*** |
| --- | --- | --- | --- | --- | --- |
|  | **MEA** | **MEB** | **MEC** |  |  |
| Phylum level |  |  |  |  |  |
| *Firmicutes* | 54.71 | 51.19 | 51.38 | 1.51 | 0.512 |
| *Bacteroidetes* | 38.81 | 43.06 | 42.54 | 1.38 | 0.395 |
| *Euryarchaeota* | 0.77 | 0.39 | 1.31 | 0.29 | 0.533 |
| *Proteobacteria* | 1.45 | 1.41 | 1.50 | 0.11 | 0.677 |
| *Gracilibacteria* | 0.51 | 0.42 | 0.59 | 0.12 | 0.613 |
| *Fibrobacteres* | 0.53 | 0.27 | 0.41 | 0.10 | 0.566 |
| *Spirochaetes* | 0.73 | 0.86 | 0.63 | 0.09 | 0.655 |
| *Tenericutes* | 1.01 | 0.74 | 0.68 | 0.07 | 0.210 |
| *Synergistetes* | 0.27 | 0.40 | 0.13 | 0.05 | 0.077 |
| *unidentified_Bacteria* | 0.31 | 0.28 | 0.24 | 0.02 | 0.403 |
| Others | 0.90 | 0.99 | 0.60 | 0.09 | 0.134 |
| Genus level |  |  |  |  |  |
| *unidentified_Bacteroidales* | 3.94 | 4.63 | 2.47 | 0.65 | 0.330 |
| *unidentified_Ruminococcaceae* | 7.31 | 6.68 | 7.36 | 0.31 | 0.403 |
| *unidentified_Lachnospiraceae* | 4.24 | 4.19 | 5.09 | 0.32 | 0.340 |
| *Saccharofermentans* | 2.75 | 2.00 | 3.51 | 0.27 | 0.085 |
| *unidentified_Prevotellaceae* | 0.67 | 0.64 | 1.26 | 0.20 | 0.733 |
| *unidentified_Rikenellaceae* | 1.63 | 1.12 | 1.18 | 0.17 | 0.878 |
| *Methanobrevibacter* | 0.72 | 0.34 | 1.27 | 0.28 | 0.482 |
| *Papillibacter* | 2.44 | 1.79 | 1.29 | 0.17 | 0.021 |
| *Succiniclasticum* | 1.44 | 1.72 | 1.97 | 0.11 | 0.137 |
| *Fibrobacter* | 0.53 | 0.26 | 0.40 | 0.10 | 0.566 |
| Others | 74.33 | 76.63 | 74.19 | 0.68 | 0.281 |

**Supplementary Table 3.** Predicted functions at level 2 of the rumen bacterial microbiota of male Hu lambs fed different dietary energy levels

| **Item** | **Groups** | | | **SEM** | ***p-value*** |
| --- | --- | --- | --- | --- | --- |
|  | **MEA** | **MEB** | **MEC** |  |  |
| Transcription | 2.76 | 2.70 | 2.70 | 0.02 | 0.460 |
| Cellular processes and signaling | 3.83 | 3.84 | 3.82 | 0.01 | 0.476 |
| metabolism | 2.44 | 2.46 | 2.43 | 0.01 | 0.534 |
| Replication and repair | 9.46 | 9.51 | 9.43 | 0.02 | 0.232 |
| Signal transduction | 1.57 | 1.54 | 1.55 | 0.01 | 0.578 |
| Infectious diseases | 0.37 | 0.37 | 0.37 | 0.00 | 0.803 |
| Lipid metabolism | 2.73 | 2.74 | 2.70 | 0.01 | 0.121 |
| Metabolic diseases | 0.11 | 0.12 | 0.11 | 0.00 | 0.779 |
| Signaling molecules and interaction | 0.15 | 0.16 | 0.16 | 0.00 | 0.363 |
| Amino acid metabolism | 10.13 | 10.21 | 10.13 | 0.03 | 0.337 |
| Metabolism of other amino acids | 1.48 | 1.51 | 1.49 | 0.01 | 0.213 |
| Metabolism of cofactors and vitamins | 4.45 | 4.47 | 4.47 | 0.01 | 0.690 |
| Nucleotide metabolism | 4.25 | 4.28 | 4.27 | 0.02 | 0.650 |
| Poorly characterized | 4.71 | 4.70 | 4.74 | 0.02 | 0.815 |
| Endocrine system | 0.31 | 0.32 | 0.31 | 0.00 | 0.316 |
| Folding sorting and degradation | 2.53 | 2.56 | 2.54 | 0.01 | 0.673 |
| Nervous System | 0.11 | 0.11 | 0.11 | 0.00 | 0.311 |
| Glycan biosynthesis and metabolism | 2.42 | 2.54 | 2.48 | 0.04 | 0.746 |
| Metabolism of terpenoids and polyketides | 1.69 | 1.71 | 1.69 | 0.01 | 0.256 |
| Cancers | 0.10 | 0.11 | 0.10 | 0.00 | 0.661 |
| Enzyme families | 2.18 | 2.20 | 2.19 | 0.01 | 0.554 |
| Digestive system | 0.04 | 0.05 | 0.05 | 0.00 | 0.368 |
| Cell motility | 2.81 | 2.66 | 2.75 | 0.07 | 0.632 |
| Xenobiotics biodegradation and metabolism | 1.53 | 1.52 | 1.50 | 0.01 | 0.169 |
| Membrane transport | 10.49 | 10.19 | 10.38 | 0.11 | 0.610 |
| Cell growth and death | 0.55 | 0.55 | 0.55 | 0.00 | 0.731 |
| Environmental Adaptation | 0.17 | 0.16 | 0.16 | 0.00 | 0.459 |
| Genetic information processing | 2.72 | 2.70 | 2.71 | 0.01 | 0.457 |
| Translation | 6.17 | 6.17 | 6.21 | 0.03 | 0.695 |
| Neurodegenerative diseases | 0.11 | 0.11 | 0.11 | 0.00 | 0.749 |
| Carbohydrate metabolism | 10.06 | 10.14 | 10.17 | 0.02 | **0.063** |
| Energy metabolism | 6.05 | 6.06 | 6.12 | 0.03 | 0.320 |
| Biosynthesis of other secondary metabolites | 1.00 | 1.02 | 1.01 | 0.01 | 0.230 |
| Immune system | 0.09 | 0.10 | 0.09 | 0.00 | 0.459 |
| Transport and catabolism | 0.34 | 0.36 | 0.34 | 0.01 | 0.562 |

**Supplementary Table 4.** The 35 most abundant KEGG pathways at level 3 in the rumen bacteria of male Hu lambs fed different dietary energy levels

| **Item** | **Groups** | | | **SEM** | ***p-value*** |
| --- | --- | --- | --- | --- | --- |
|  | **MEA** | **MEB** | **MEC** |  |  |
| Pyrimidine metabolism | 2.01 | 2.02 | 2.01 | 0.01 | 0.533 |
| Energy metabolism | 1.03 | 1.04 | 1.03 | 0.01 | 0.619 |
| Function unknown | 1.15 | 1.15 | 1.18 | 0.01 | 0.543 |
| Homologous recombination | 1.02 | 1.03 | 1.02 | 0.00 | 0.145 |
| Transcription factors | 1.48 | 1.44 | 1.44 | 0.02 | 0.756 |
| Two component system | 1.43 | 1.40 | 1.41 | 0.02 | 0.619 |
| Secretion system | 1.22 | 1.19 | 1.21 | 0.01 | 0.249 |
| Chromosome | 1.62 | 1.63 | 1.62 | 0.00 | 0.763 |
| Ribosome biogenesis | 1.48 | 1.48 | 1.48 | 0.00 | 0.970 |
| ABC transporters | 2.84 | 2.74 | 2.80 | 0.04 | 0.527 |
| Transporters | 5.61 | 5.45 | 5.55 | 0.07 | 0.651 |
| DNA replication proteins | 1.35 | 1.36 | 1.35 | 0.00 | 0.468 |
| Purine metabolism | 2.24 | 2.25 | 2.26 | 0.01 | 0.691 |
| Aminoacyl tRNA biosynthesis | 1.25 | 1.24 | 1.26 | 0.01 | 0.336 |
| Arginine and proline metabolism | 1.29 | 1.30 | 1.28 | 0.00 | 0.038 |
| Phenylalanine tyrosine and tryptophan biosynthesis | 0.93 | 0.94 | 0.96 | 0.00 | 0.009 |
| Oxidative phosphorylation | 1.23 | 1.25 | 1.23 | 0.01 | 0.763 |
| DNA repair and recombination proteins | 2.98 | 3.00 | 2.97 | 0.01 | 0.230 |
| Starch and sucrose metabolism | 0.97 | 0.99 | 1.00 | 0.01 | 0.619 |
| Bacterial motility proteins | 1.26 | 1.19 | 1.24 | 0.03 | 0.527 |
| Amino acid related enzymes | 1.56 | 1.57 | 1.57 | 0.01 | 0.677 |
| Other ion coupled transporters | 1.05 | 1.06 | 1.04 | 0.00 | 0.185 |
| Pyruvate metabolism | 1.05 | 1.05 | 1.04 | 0.00 | 0.181 |
| Translation proteins | 0.98 | 0.98 | 0.98 | 0.00 | 0.878 |
| Alanine aspartate and glutamate metabolism | 1.12 | 1.14 | 1.13 | 0.00 | 0.221 |
| Amino sugar and nucleotide sugar metabolism | 1.37 | 1.38 | 1.39 | 0.00 | 0.121 |
| Ribosome | 2.64 | 2.65 | 2.66 | 0.01 | 0.613 |
| Peptidases | 1.92 | 1.94 | 1.93 | 0.01 | 0.379 |
| Glycolysis/Gluconeogenesis | 1.07 | 1.07 | 1.08 | 0.00 | 0.114 |
| General function prediction only | 3.56 | 3.55 | 3.56 | 0.01 | 0.756 |
| Cysteine and methionine metabolism | 1.01 | 1.00 | 1.00 | 0.00 | 0.379 |
| Transcription machinery | 1.10 | 1.08 | 1.07 | 0.00 | 0.015 |
| Methane metabolism | 1.37 | 1.33 | 1.42 | 0.03 | 0.298 |
| Carbon fixation pathways in prokaryotes | 1.11 | 1.11 | 1.10 | 0.01 | 0.827 |
| Chaperones and folding catalysts | 1.03 | 1.05 | 1.04 | 0.01 | 0.691 |
